# Chlorine, chromium, proteins of oxidative stress and DNA repair pathways are related to prognosis in oral cancer

**DOI:** 10.1038/s41598-021-01753-x

**Published:** 2021-11-16

**Authors:** Aricia Leone Evangelista Monteiro de Assis, Anderson Barros Archanjo, Raul C. Maranhão, Suzanny O. Mendes, Rafael P. de Souza, Rafael de Cicco, Mayara M. de Oliveira, Aline R. Borçoi, Lucas de L. Maia, Fabio D. Nunes, Marcelo dos Santos, Leonardo O. Trivilin, Christiano J. G. Pinheiro, Adriana M. Álvares-da-Silva, Breno Valentim Nogueira

**Affiliations:** 1 Biotechnology Graduate Program/RENORBIO, Federal Univerty of Espírito Santo, Vitória, 29040090 Brazil; 2 Heart Institute (InCor), Medical School Hospital, University of São Paulo, São Paulo, 05403900 Brazil; 3 Cancer Institute Arnaldo Vieira de Carvalho, São Paulo, 01219010 Brazil; 4 Department of Stomatology, Faculty of Dentistry, University of São Paulo, São Paulo, 05508000 Brazil; 5 Multicampi School of Medical Sciences of Rio Grando Do Norte, Federal University of Rio Grande Do Norte, Caicó, 59300000 Brazil; 6 Department of Veterinary Medicine, Center for Agricultural Sciences and Engineering, Federal University of Espírito Santo, Alegre, 29500000 Brazil; 7 Department of Rural Engineering, Center for Agricultural Sciences and Engineering, Federal University of Espírito Santo, Alegre, 29500000 Brazil; 8 Department of Morphology, Health Sciences Center, Federal University of Espírito Santo, Vitória, 29047105 Brazil

**Keywords:** Biotechnology, Chemical biology, Cancer, Cancer metabolism, Cancer prevention, Cancer screening, Oral cancer, Tumour biomarkers

## Abstract

The comparison of chemical and histopathological data obtained from the analysis of excised tumor fragments oral squamous cell carcinoma (OSCC) with the demographic and clinical evolution data is an effective strategy scarcely explored in OSCC studies. The aim was to analyze OSCC tissues for protein expression of enzymes related to oxidative stress and DNA repair and trace elements as candidates as markers of tumor aggressiveness and prognosis. Tumor fragments from 78 OSCC patients that had undergone ablative surgery were qualitatively analyzed by synchrotron micro-X-ray fluorescence for trace elements. Protein expression of SOD-1, Trx, Ref-1 and OGG1/2 was performed by immunohistochemistry. Sociodemographic, clinical, and histopathological data were obtained from 4-year follow-up records. Disease relapse was highest in patients with the presence of chlorine and chromium and lowest in those with tumors with high OGG1/2 expression. High expression of SOD-1, Trx, and Ref-1 was determinant of the larger tumor. Presence of trace elements can be markers of disease prognosis. High expression of enzymes related to oxidative stress or to DNA repair can be either harmful by stimulating tumor growth or beneficial by diminishing relapse rates. Interference on these players may bring novel strategies for the therapeutic management of OSCC patients.

## Introduction

Oral squamous cell carcinoma (OSCC) is the most common type of oral cavity cancers. These cancers predominanly occur between the fifth and the seventh decades of life and are the sixth most prevalent cancer type in the world population^1^. Despite the improvement in diagnostic and therapeutic procedures, tumor relapse after surgical removal is frequent and the overall 5-year survival rates are low^2–4^. Smoking is the chief risk factor for OSCC, followed by alcoholic addiction. HPV infection and genetic susceptibility are other OSCC risk factors^5^.

For the first time our group demonstrated the presence of important trace elements and non-essential or toxic elements in OSCC samples, suggesting a fundamental relationship between smoking and the presence of certain elements. In addition, we have shown that the presence of the elements manganese and chloride have proven to be important prognostic and survival factors for patients with head and neck cancer^6^.

It has now been well established that non-essential metals and metaloids can induce carcinogenicity by favoring the generation of reactive oxygen species (ROS). The formation of metal-mediated free radicals can cause several modifications in DNA bases, increases lipid peroxidation, and elicits alterations in the body homeostasis of calcium and sulfhydryl^7^.

Unbalance between ROS production and degradation leads to oxidative stress that may participate in the induction of inflammation and carcinogenesis^8^. Enzymes involved in the protection against damage from oxidative stress, such as superoxide dismutase (SOD-1), thioredoxin (Trx), and in DNA repair, such as purinic/apyrimidinic endonuclease/redox factor-1 (Ref-1) and 8-oxoguanine glycosylase (OGG) are important natural defenses against cancer development. Those enzymes may eventually be used as targets for acquisition of prognostic markers and therapeutic strategies^9^.

SOD-1 catalyzes the dismutation of superoxide radical into oxygen and hydrogen peroxide (H_2_O_2_)^9^. In the intracellular medium, metals react with H_2_O_2_ and generate hydroxyl radical (OH^−^) capable of acting against direct damages to DNA. Damaged DNA can be repaired by the action of OGG1 glycosylase, which excises the modified base^10^. Ref-1 protects the DNA structure against enzymatic degradation while the specific enzymes complete the repair^11^. The activity of Ref-1 is regulated by Trx^11,12^.

Since OSCC frequently relapses after surgery, it is of prime importance to determine the factors that contribute for the worse prognosis of those patients. In this study, we hypothesize whether differences in content in the tumoral tissue of metals and metaloids and in the protein expression of enzymes related with DNA repair and oxidative stress could be related to disease relapse. Therefore, the aim was to analyze OSCC tissues for protein expression of enzymes related to oxidative stress and DNA repair and trace elements as candidates as markers of tumor aggressiveness and prognosis.

## Results

### Relationship between epidemiological, clinicopathological characteristics, trace elements and protein expression

Patients aged over 63 years were sevenfold less likely to relapse than those below this age cut-off (p < 0.05). In addition, we also observed that patients consuming alcoholic bevarages were eightfold more likely to relapse (p < 0.05) and sixfold more likely to die from the disease than those that did not consume alcohol (p < 0.01). Also, in Table 1, patients with tumors with vascular invasion were 13-fold more likely to relapse (p < 0.01) and sixfold more likely to die (p < 0.05) as compared with those with tumors without vascular invasion. Perineural invasion was not related to disease relapse, but death rates were increased fourfold in patients with tumors with this feature (p < 0.05).

**Table 1:** Logistic regression model between prognostic, epidemiological and clinicopathological characteristics, trace elements and protein expression.

Features	Logistic regression model	
	Relapse	Death
**Age**		
> 63/≤ 63^a^		
OR	0.135	–
CI 95%	0.022–0.812	
p value	**0.029**	
**Current alcohol consumption**		
Yes/no^a^		
OR	8.437	6.391
CI 95%	1.098–64.806	1.672–24.422
p value	**0.040**	**0.007**
**Vascular invasion**		
Present/absent^a^		
OR	13.516	6.072
CI 95%	1.900–96.129	1.276–28.902
p value	**0.009**	**0.023**
**Perineural invasion**		
Present/absent^a^		
OR		4.054
CI 95%	–	1.00–16.443
p value		**0.050**
**Chlorine**		
Present/absent^a^		
OR	0.076	–
CI 95%	0.007–0.827	
p value	**0.034**	
**Chrome**		
Present/absent^a^		
OR	8.003	–
CI 95%	1.315–48.709	
p value	**0.024**	
**OGG1/2 cytoplasmic**		
High/low^a^		
OR	0.041	
CI 95%	0.004–0.414	–
p value	**0.007**	
**SOD-1 cytoplasmic**		
High/low^a^		
OR	–	2.592
CI 95%		0.458–14.654
p value		0.281
**Trx cytoplasmic**		
High/low^a^		
OR	–	1.972
CI 95%		0.568–6.841
p value		0.285

*OR* odds ratio, *CI* confidence interval. Values in bold represent p < 0.05.

^a^Reference variable.

Fifteen trace elements were identified in the different tumor fragments analysed by microXRF, namely iron and zinc (present in 100% of the tumor fragments), sulfur and calcium (99%), copper (95%), phosphorus (94%), potassium (83%), arsenic and bromine (68%), chromium (43%), chlorine (36%), manganese (33%), nickel (14%), magnesium and cobalt (12%). The finding of chlorine in the tumor fragments was determinant of reduction of frequency of disease relapse in the patients by 13-fold, as compared to tumor fragments without detectable chlorine (p < 0.05). In contrast, the finding of chromium presence in the tumor fragment was determinant of an eightfold increase in the % of disease relapse (p < 0.05, Table 1).

**Figure 1:**
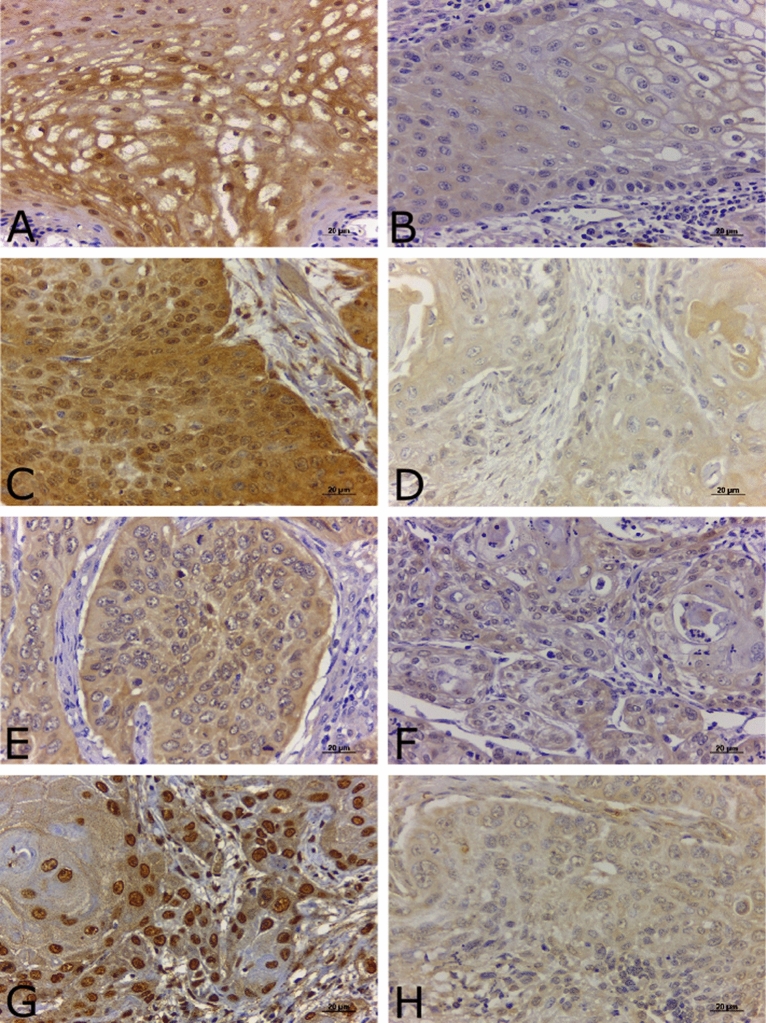
Imunnohistochemistry photomicrographs. (**A**) Strong intensity cytoplasmic and nuclear immunostaining of SOD-1. (**B**) Low intensity cytoplasmic immunostaining of SOD-1. (**C**) Strong intensity cytoplasmic and nuclear immunostaining of OGG1/2. (**D**) Low intensity cytoplasmic immunostaining of OGG1/2. (**E**) Strong intensity cytoplasmic immunostaining of Trx. (**F**) Low intensity cytoplasmic immunostaining of Trx. (**G**) Strong intensity cytoplasmic and nuclear immunostaining of Ref-1. (**H**) Low intensity cytoplasmic immunostaining of Ref-1. *SOD-1* superoxide dismutase, *Ref-1* purinic/apyrimidinic endonuclease/redox factor-1, *OGG1/2* 8-oxoguanine glycosylase. Original magnifications of ×400.

Also shown in Table 1, all four proteins analysed here by immunohistochemistry were expressed in both nucleous and cytoplasm of the tumor cells (Fig. 1). In patients with tumors with high expression of OGG1/2, the frequency of relapse was reduced by twenty-fourfold as compared to those with low expression of this protein (p < 0.01). Regarding the other three proteins, namely Trx, SOD-1 and Ref-1, no correlations were found with relapse or death rates.

In Table 2 it is shown that high cytoplasmatic expressions of Trx, and SOD-1 and high nuclear expression of Ref-1 were associated with larger tumor sizes. High cytoplasmic expression of Trx (p < 0.05) and SOD-1 (p < 0.05) were related to three and to fivefold larger tumor sizes, respectively. The high nuclear expression of Ref-1 was associated to 6-fold larger tumor size (p < 0.05).

**Table 2:** Logistic regression model concerning lymph node, tumor size, aggressiveness and vascular invasion with epidemiological and clinicopathological characteristics, protein expression and trace elements.

Features	Logistic regression model			
	Lymph node	Tumor size	Aggressiveness	Vascular invasion
**Smoking**				
Yes/no^a^				
OR	3.38	–	–	–
CI 95%	0.81–14.09			
p value	0.093			
**Tumor size**				
≥ pT3/≤ pT2^a^				
OR	–	–	–	2.31
CI 95%				0.62–8.58
p value				0.211
**Vascular invasion**				
Present/absent^a^				
OR	47.16	–	15.67	–
CI 95%	8.35–266.17		1.49–164.3	
p value	**< 0.001**		**0.022**	
**Perineural invasion**				
Present/absent^a^				
OR	–	–	–	10.30
CI 95%				2.74–38.60
p value				**0.001**
**Trx nuclear**				
High/low^a^				
OR	1.14	–	–	–
CI 95%	0.256–5.11			
p value	0.861			
**SOD-1 cytoplasmic**				
High/low^a^				
OR	–	4.63	–	2.81
CI 95%		1.12–19.08		0.28–28.34
p value		**0.034**		0.380
**Trx cytoplasmic**				
High/low^a^				
OR	–	3.00	–	–
CI 95%		1.09–8.24		
p value		**0.033**		
**Ref-1 nuclear**				
High/low^a^				
OR	–	6.12	–	–
CI 95%		1.14–32.83		
p value		**0.034**		
**Potassium**				
Present/absent^a^				
OR	–	–	0.15	–
CI 95%			0.01–2.04	
p value			0.158	

*OR* odds ratio, *CI* confidence interval. Values in bold represent p < 0.05.

^a^Reference variable.

In Table 2, we also found by multivariate analysis that the lymph node vascular involvement was increased by 47-fold (p < 0.001) when tumors had vascular invasion. There was a trend for lymph node involvement in tumors of smokers that was not statistically significant (p = 0.09). The vascular invasion in tumors also increased the tumor aggressiveness by 15-fold (p < 0.05), whereas the presence of perineural invasion increased the vascular invasion by tenfold (p < 0.001). No association was found between tumor size and tumor vascular invasion (p = 0.211).

### Survival in patients which oral squamous cell carcinoma

**Figure 2:**
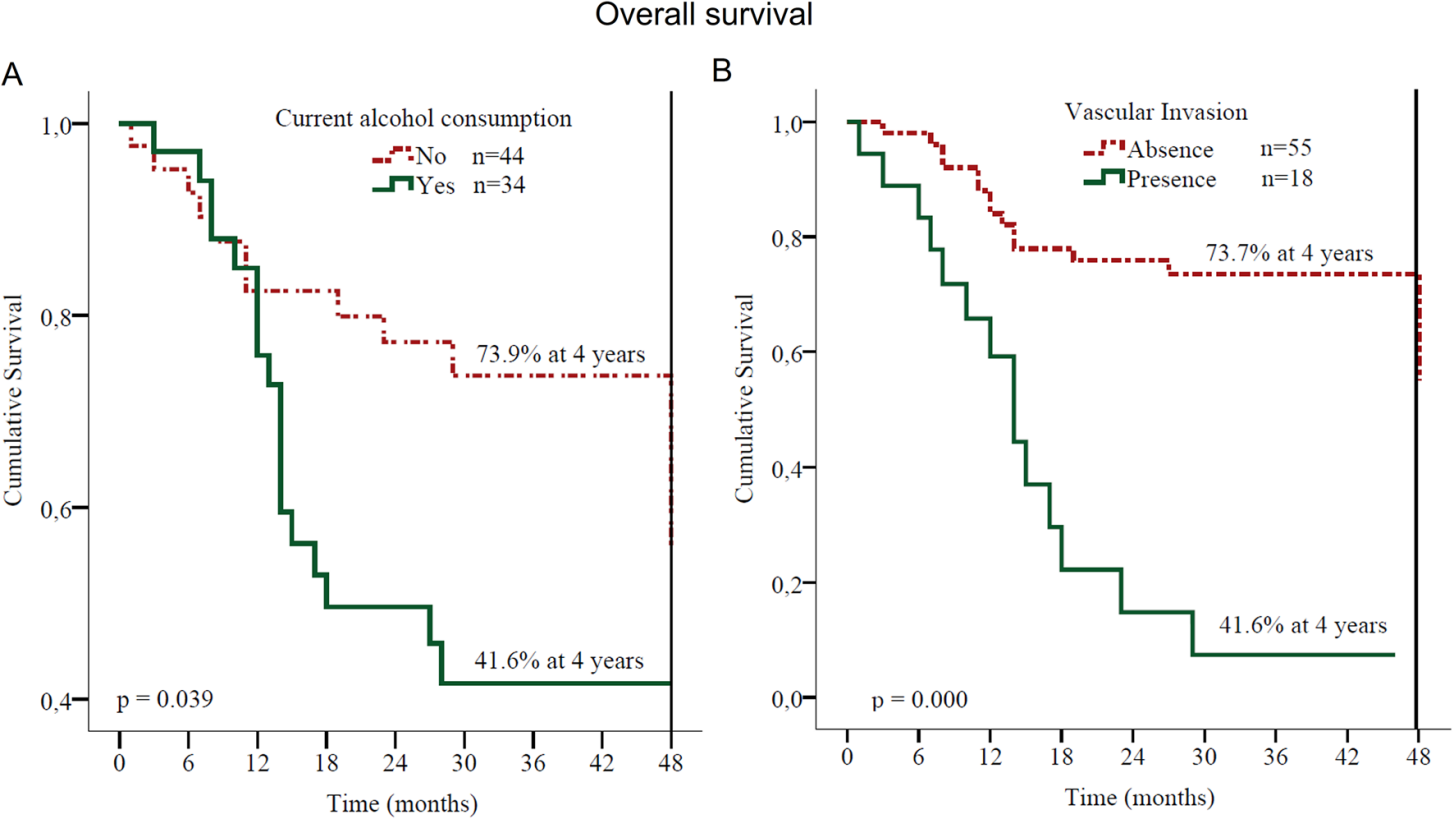
Overall survival plot. Kaplan–Meier curve is shown for overall survival in patients with squamous cell carcinoma of the oral cavity according to (**A**) current alcohol consumption and (**B**) vascular invasion.

In Table 3 it is shown that current alcohol consumption is a risk factor for shorter relapse-free survival and increased threefold the probability of disease relapsing (p < 0.05; Table 3). Current alcohol consumption also decreased the overall survival (p < 0.05). The four-year survival after surgery was only 41.6% in alcohol consumption and 73.9% in non-alcohol addicted (Fig. 2A). The multivariate analysis also showed that alcohol consumption is a risk factor for shorter overall survival, with twofold increase in risk (p < 0.05; Table 3).

**Table 3:** Cox model of prognostic factors and survival in patients which oral squamous cell carcinoma.

Features	Cox model	
	Recurrence-free survival	Overall survival
**Current alcohol consumption**		
Yes/no^a^		
HR	3.247	2.370
CI 95%	1.022–10.135	1.035–5.423
p value	**0.046**	**0.041**
**Vascular invasion**		
Present/absent^a^		
HR	5.108	2.954
CI 95%	1.536–16.989	1.164–7.499
p value	**0.008**	**0.023**
**Perineural invasion**		
Present/absent^a^		
HR		1.871
CI 95%	–	0.730–4.793
p value		0.192
**Chlorine**		
Present/absent^a^		
HR	0.210	
CI 95%	0.046–0.970	–
p value	**0.046**	
**Chrome**		
Present/absent^a^		
HR	2.980	–
CI 95%	0.917–9.688	
p value	0.069	
**Manganese**		
Present/absent^a^		
HR	–	1.548
CI 95%		0.710–3.375
p value		0.272
**Trx cytoplasmic**		
High/low^a^		
HR	2.019	–
CI 95%	0.632–6.450	
p value	0.236	

*HR* hazards ratio, *CI* confidence interval. Values in bold represent p < 0.05.

^a^Reference variable.

Four years after surgery, 58.4% of patients with tumor vascular invasion died, as compared with 26.3% of those whose tumors did not have vascular invasion (Fig. 2B). Multivariate analysis showed that tumor vascular invasion was a risk factor for shorter overall survival, increasing threefold the probability of death (HR = 2.954, CI 1.164–7.499; Table 3).

**Figure 3:**
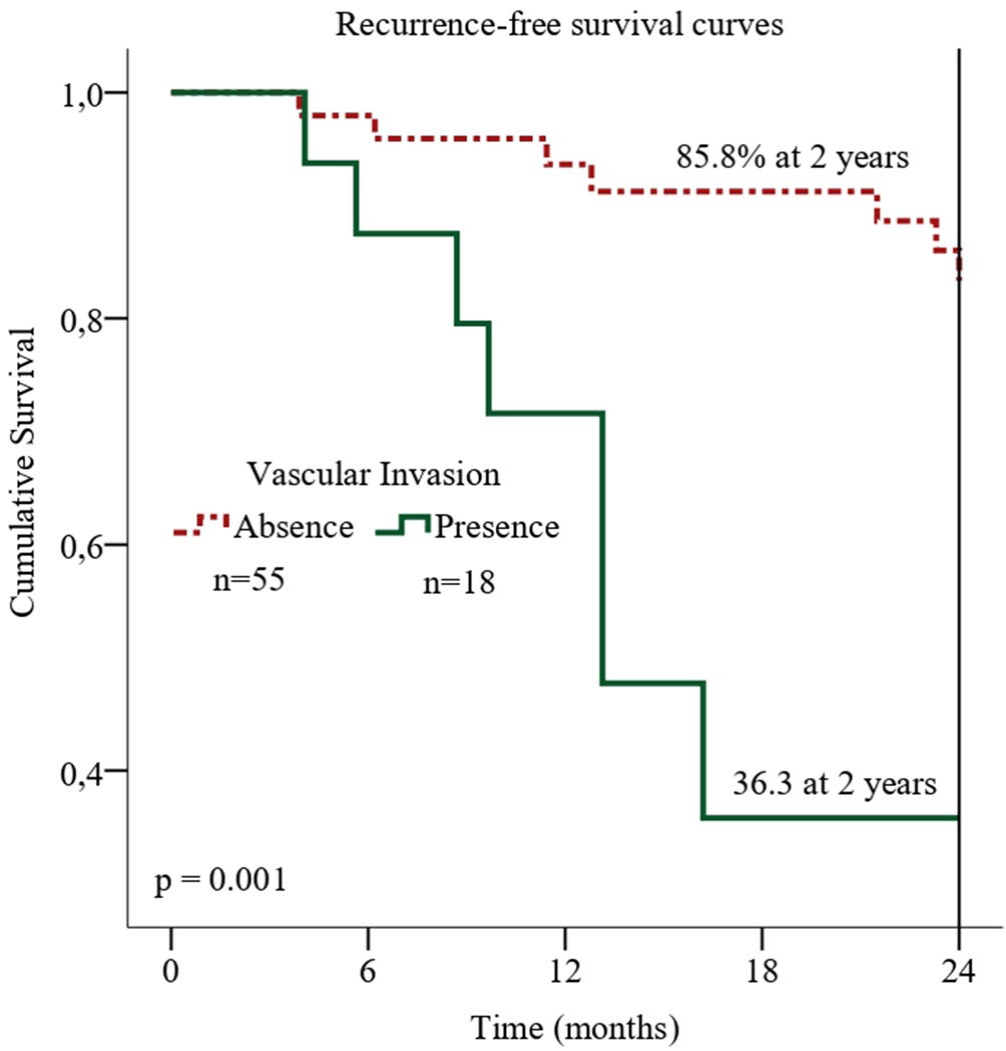
Recurrence-free survival plot. Kaplan–Meier curve is shown for recurrence-free survival in patients with squamous cell carcinoma of the oral cavity according to vascular invasion.

Patients with tumors with vascular invasion had lower probability of relapse-free survival (p < 0.01): within two years after surgery, 63.7% of patients with vascular invasion had disease relapse, as compared with 14.2% of those with tumors without vascular invasion (Fig. 3). In the multivariate analysis, tumor vascular invasion appeared as fivefold increased risk for shorter disease-free survival (HR = 5.108, CI 1.536–16.989; Table 3).

In Table 3 it is also shown that the presence in the tumor of chlorine was associated with fivefold decrease in the disease relapse rates (HR = 0.210, CI 0.046–0.970). There was a trend not statiscally confirmed (p = 0.069) that presence of chromium would be related to shorter relapse-free survival.

## Discussion

The results of this study respecting the relationships between the histopathological characteristics of OSCC tumors and the data of the clinical evolution of the patients were confirmatory of the reports from the literature^13–19^. In several previous studies, the presence of vascular and perineural invasion of the tumor has been related with worse prognosis, i.e., disease relapse, shorter survival and higher death rates^17,19^. In fact, the vascular and perineural invasion of the tumor are fundamental mechanisms for metastatization and recurrence of tumors and constitute important prognostic factors^14–16^. Regarding the demographic relationships, our results are also in agreement with the previous reports of older age being a factor for better prognosis^20,21^. The classical relation between alcohol consumption and worse prognosis^22–25^ was also documented here. Unexpectedly, in contrast with previous reports^26–28^ the smoking habit was not determinant of worse prognosis. It is possible that, in our study, the fact that among non-smokers few had never smoked, and many were ex-smokers, has concealed this rather classical relationship. Therefore, in general terms, the Brazilian population sample studied here had the typical prognostic features of those from other countries.

Our research group was the first to investigate the presence of metals in oral cancer by µ-XRF analysis^6^. Using this simple and straightforward approach, it is possible to perform multielemental analysis without the disruptive preparation of the tissue samples. It is of note that among the fifteen different metals analysed in the tumor fragments only two had relationship with the disease evolution data of the patients. The presence of two of those two metals, chlorine and chromium was associated to disease relapse.

In the patients studied here, the concentration in the serum of the metals was not determined. Nonetheless, in none of the previous studies with elevated serum chlorine was found in oral cancer patients, in contrast, high concentrations of Cu and Zn were reported in patients with this cancer type but with no relationship with the clinical evolution or demographic data^29–31^. Our finding that the presence of chromium in the tumor was determinant of worse clinical evolution and that of chloride of better evolution suggests that the analysis of the tumor metal content may be important to unravel new mechanisms underlying the course of the disease. The evolution of techniques that can be used in the clinical laboratory to determine chromium and chloride in OSCC tumor tissues may offer an interesting tool to routinely evaluate the prognosis of those patients as based on the presence of those elements. These consistent relationships of the two metals and prognosis found here can also make grounds for noval therapeutic targets.

Some hints of mechanisms whereby the presence of chromium may adversely affect the clinical evolution, with increased relapse rates, can be suggested by the studies of Shi et al.^32,33^. Those authors postulate that reduction of hexavalent Cr to trivalent Cr generates oxygen radicals with activation of signaling pathways of apoptosis inhibition, via PI3K and AKT. The inhibition of apoptosis leads to the accumulation of mutations. This favors microenvironmental changes that stimulate tumor progression and relapse.

In respect to our finding of the relationship between presence of chlorine and decreased rate of tumor recurrence, it is possible that excess chlorine consequent to disturbances in the ionic channel function, specifically the chloride intracellular chanell 1 e 4 (CLIC1 CLIC4), lowers the cytoplasmic pH thereby inducing the tumor cell apoptosis. In fact, it has been recently shown that CLIC1 is involved in the regulation of the cell cycle and of cell volume, as well as in the regulation of apoptosis. It is postulated that CLIC1 has an important role in tumor development^6,34,35^.

Regarding the protein expression of the four different enzymes studied here, only OGG1/2 expression showed relation with disease prognosis. Nonetheless, the expression of the three other enzymes, SOD1, Ref-1 and Trx were related to the tumor size. In patients with tumors exihibiting high expression of OGG1/2, there was less occurrence of disease relapse.

The protective action against disease relapse offered by high OGG1/2 expression in the tumors can be ascribed to the reduction of ROS and of mitochondrial DNA damage resulting from the action of this enzyme. OGG1/2 inhibits the activation of p-AKT and of HIF1 which leads to decrease in progression and metastatization of tumors, as observed in mice models of breast tumor^36^. In OGG1/2 KO mice, ROS accumulation occurred, together with non-repair of oxidative damage generated in DNA by suppressing the Nrf2 pathway^37^. Progression of hepatocellular adenocarcinoma induced by phenobarbital to hepatocellular carcinoma was increased in the KO animals, which highlights the importance of OGG1/2 as a key enzyme acting in DNA repair^37^. Thus, our finding that disease relapse was less frequent in patients with high OGG1/2 expression was in line with those anti-neoplastic actions of this enzyme.

Noteworthy was the fact that the expression of the three other enzymes that were unrelated to disease progression had otherwise high relation with the size of the tumors. In this respect, high nuclear expression of Ref-1 determined sixfold larger tumors, while cytoplasmic high expression of SOD-1 and Trx were related to four and threefold larger tumors, respectively. The association between tumor size and the high expression of these enzymes can be accounted for the activities of the enzymes that favor tumor growth. SOD-1 increases the oxidative burden, Ref-1 activates transcription factors such as early-response protein-1 (Egr-1), NF-κB, p53, HIF1α (AP-1), which are involved in various cellular processes, including cell survival and growth^38–41^. Trx-1 expression has been related to cancer development and spread^42^. Trx-1 has redox activity and is related to activation of different transcription factors of inflammation regulation, including NF-kB and activator protein-1 (AP-1)^12^. In addition, its action may increase expression of HIF1α, a hypoxia transcription factor^43^, possibly by inhibiting the degradation of HIF1α^44^. Trx-1 binds and inhibits pro-apoptotic proteins, including apoptosis signal by regulating kinase-1 (Ask-1)^45^. Thus, increased protein expression of Trx-1 suggests that increase in the activity of this enzyme would favor tumor growth. This may occur by either altering inflammatory factors, or by activation of angiogenesis via HIF-VEGF.

The results show that detectable amounts chromium and chlorine in the tumor tissue, as well as the occurrence of high protein expression of OGG1/2, SOD1, Ref-1 and Trx-1 may influence the tumor growth and predict the clinical evolution of OSCC. These findings, obtained by an innovative methodological approach, may lead to the establishment of useful tools not only for disease prognosis but eventually for acquisition of new therapeutic targets.

The acquisition of practical techniques that can be used in the clinical laboratory to determine chromium and chloride in OSCC tumoral tissues may bring the current results to the routine Oncology practice.

## Methods

### Ethics approval and consent to participate

This study was approved by the Committees of Ethics Research of the Federal University of Espírito Santo [CAAE: 49091515.9.0000.5060] and the Arnaldo Vieira de Carvalho Cancer Institute [CAAE: 49091515.9.3002.5471]. The study was carried out in accordance with all relevant guidelines. As this was a retrospective study, the requirement for informed consent was waived both by the Committees of Ethics Research of the Federal University of Espírito Santo and by the Ethics Committee of the Arnaldo Vieira de Carvalho Cancer Institute. The GENCAPO project was approved by National Research Ethics Commission (CONEP) [Technical advice: 128/2012; CONEP: 16491].

### Study subjects

Tumor samples, demographic, hystopathological and clinical evolution data were obtained from 78 patients with hystopathological diagnosis of oral cavity squamous cell carcinoma (Table 4). Samples from patients of both sexes, older than 18 years, smokers and non-smokers who were diagnosed with squamous cell carcinoma of the head and neck treated by the Arnaldo Vieira de Carvalho Cancer Institute (ICAVC) in São Paulo, Brazil, between January 2012 and May 2015. The follow-up period was 4 years. They were participants of an ongoing Genome Head and Neck Project (GENCAPO), involving a multi-institutional and multidisciplinary group that has been active since 2002. Exclusion criteria were: oropharyngeal cancer, previous surgical or chemotherapy treatment, presence of distant metastasis, no removal of cervical lymph nodes and positive surgical margins. Tumor fragments inbedded in paraphin blocks were used for the analyses of protein and elementary characterization performed in this study. The variables tumor size (pT) and lymph node status (N) in the following associations were considered to define the tumor aggressiveness variable: pT3/pT4 (N0) for “Less aggressive” and pT1/pT2 (N1) for “More aggressive”.

**Table 4:** Epidemiological, clinicopathological and prognostic characteristics of patients with oral squamous cell carcinoma.

Characteristics	Total	
	N	(%)
**Gender**		
Female	24	30.77
Male	54	69.23
**Age (years)**		
Mean	63.69	
Standard deviation	± 11.01	
Median	63	
Minimum	45	
Maximum	87	
**Age (category)**		
≤ 63 years	41	52.60
> 63 years	37	47.40
**Smoking**		
Never	14	17.95
Yes, in the past	23	29.49
Yes, currently	41	52.56
**Consumption of alcoholic beverages**		
Never	17	21.79
Yes, in the past	27	34.62
Yes, currently	34	43.59
**Tumor size (pT)**^a^		
pT1	25	32.10
pT2	19	24.40
pT3	17	21.80
pT4	17	21.80
**Lymph node (pN)**^a^		
Negative	52	66.66
Positive	26	33.34
**Vascular invasion**		
Absent	55	70.50
Present	18	23.00
Not evaluable^b^	5	6.50
**Aggressiveness**		
Less aggressive^c^	18	23.08
More aggressive^d^	14	17.95
Other categories^b^	46	58.97
**Perineural invasion**		
Absent	43	55.13
Present	27	34.62
Not evaluable^b^	8	10.26
**Recurrence**		
No	63	80.77
Yes	15	19.23
**Death due to ilness**		
No	50	64.11
Yes	28	35.99
Total	78	100.00

*pT* tumor size, *pN* lymph node.

^a^TNM Classification (7th edition).

^b^Did not enter in statistical calculations.

^c^pT3/pT4 (N0).

^d^pT1/pT2 (N1).

### Tissue microarrays

Tissue microarrays of the tumor fragments were made as previously described^46^, with selection of two representative tumor areas, as evaluated by two experienced pathologists from tissue slides stained with hematoxylin/eosin, followed by extraction of two 1.5 mm diameter cylinders from each sample which were then added to the microarray receptor block using a tissue microarrayer (BEECHER INSTRUMENTS, Silver Spring, MD, USA). Sections were then removed from the TMA and mounted on microscopy slides. A pathologist checked the content of each spot. Spots bent or missing more than 70% of the tissue were excluded.

### Qualitative elementary characterization

**Figure 4:**
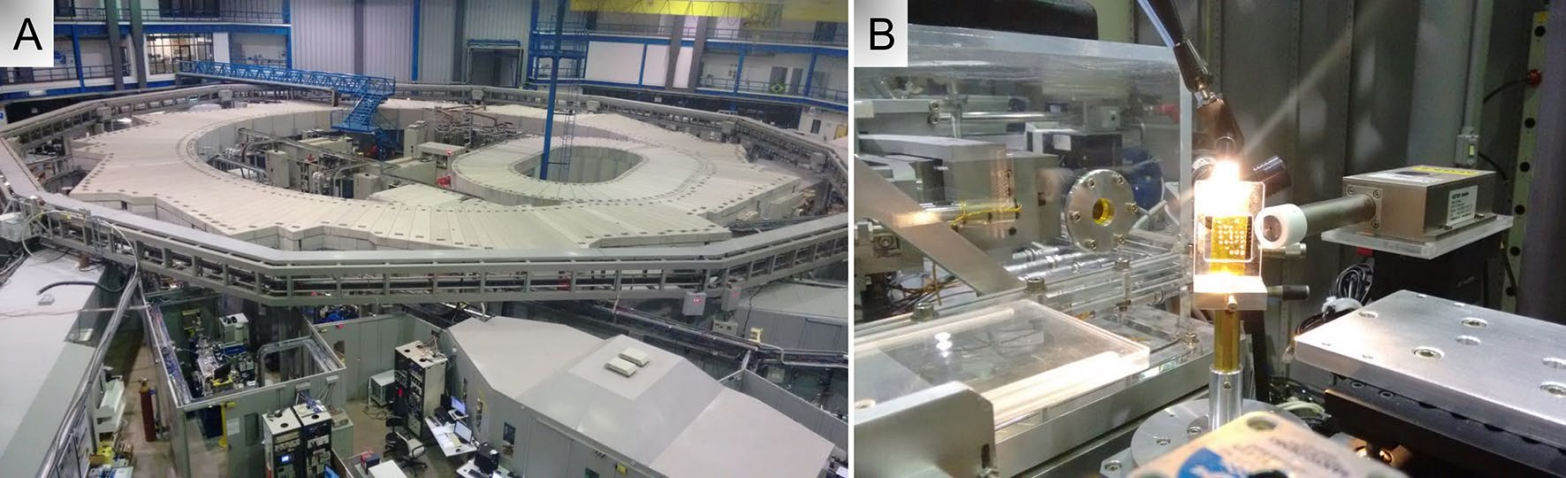
Cyclic accelerator of synchrotron light. (**A**) The synchrotron micro-X-ray fluorescence (µ-XRF) radiation was used to detect chemical elements from electron excitation energy absorption performed on the D09-XRF beamline in oral cancer samples, at the Brazilian Synchrotron Light Laboratory (LNLS), Campinas, SP, Brazil. (**B**) The samples they were deposited in plastic support with ultralene film the synchrotron radiation-based μ-XRF technique was used to detect chemical elements. Using a camera attached to an optic microscope it was possible to determine the point of incidence of the beam on the sample. Nine measurements in a 3 × 3 matrix were performed.

For the qualitative elemental characterization, tumor tissue samples (mean thickness of 450 μm; mean density of 0.54 g/cm^3^) were removed from the TMA and subjected to dewaxing and rehydration processes with xylene, alcohol (the quality and integrity of these reagents were checked or purity in each batch used) and ultrapure water. They were then deposited in plastic support with Ultralene film (SPEX SAMPLEPREP, Metuchen, NJ, USA) and sent to the D09-XRF beamline equipment. For the elementary characterization, the synchrotron radiation-based μ-XRF technique was used to detect chemical elements from electron excitation energy absortion, which is specific for each element. To obtain the spectra, a white beam with a power range of 4–24 keV and dimensions of 2 mm^2^ was applied to the samples for 20 s and excited the eletons. Nine measurements in a 3 × 3 matrix were performed and later an averaging was performed to obtain the final spectrum used in analyzes. For adjustment of characteristic X-ray spectra, determination of elements and their respective fluorescent intensities, an analysis of a certified reference sample was carried out, Standard Reference Material^®^ 1577b “Bovine Liver”, produced by National Institute of Standards and Technology (Gaithersburg, MD, USA), under the same conditions as the test samples, PyMca 5.0.0 software program^6,47^ was the basis for analyzes. Measurements of μXRF were performed in D09-X-Ray Fluorescence (D09-XRF) light line at the National Synchrotron Light Laboratory (Fig. 4), Campinas, São Paulo, Brazil^48^. Spectra were obtained by the average of nine measured points and analysis was peformed in program PyMca 5.0.0. Each spectrum was verified, the characteristic peaks of the elements identified, and then identification values were assigned as (0) for absence and (1) for the presence of the characteristic peak.

### Immunohistochemistry

For the standardization of the immunohistochemistry analyses, samples were collected from more interiorized regions of the tumors, always giving priority to the presence of tumor cells, thus avoiding margins, regions of necrosis and inflammatory infiltrates, as they influence the expression of proteins. Antibodies anti-SOD-1 (SC-11407, Santa Cruz Biotechnology) was diluted 1:100 with Antibody Diluent (Catalog no. ADS-125, Spring Bioscience, Canada), anti-Ref-1 was diluted 1:400 (SC-17774, Santa Cruz), anti-OGG1/2 was diluted 1:100 (SC-376935, Santa Cruz Biotechnology) and anti-Trx was diluted 1:100 (SC-166393, Santa Cruz Biotechnology) were used in immunohistochemistry reaction with REVEAL Polymer-HRP (Spring Bioscience), according to the manufacturer's protocol. For each reaction negative controls (absence of primary and secondary antibody) were used. Protein expression was independently evaluated by two different observers, and conflicting results were submitted to re-analysis. Protein analysis was semiquantitative so that samples were classified according to % of cells stained at: 0 (0% of labeled cells), 1 (< 10%); 2 (10 ≤ 50%) and 3 (> 50% of labeled cells); and by staining intensity in: 0 (negative), 1 (weak), 2 (moderate) and 3 (strong). Scores estimated from the % and intensity of staining were multiplied and their means calculated for each sample. On the basis of the final score, each sample was cathegorized as negative (0), weak positive (1 ≤ 3) or strong positive (> 3), according to method used by studies that performed similar analyzes to the present study^49,50^.

### Statistical analysis

For association tests, Chi-square test was used in bivariate analysis and, when necessary, Fisher's exact test, with a 5% margin of error and with Bonferroni correction. Multivariate logistic regression by modeling was used to adjust odds ratio (OR) and confidence interval (CI ≥ 95%). The variables that obtained a p-value of less than 20% (p < 0.20) were inserted by the backward method in multivariate logistic regression model, with the significant variables remaining at the end of the model, at each stage (p < 0.05). For overall survival analysis, it was calculated the time interval (in months) between dates of surgery and death by disease of each patient or the last return in cases of survivors. The time interval for recurrence-free survival analysis was calculated using as end points the dates of global relapse, or the date of the last return in asymptomatic cases. The Kaplan–Meier model was used for survival analysis, using the Wilcoxon p-value and the Cox proportional hazards to adjust p-values, and to hazards ratio (HR) and CI (CI ≥ 95%). The values of OR and HR were adjusted for lymph node status (TNM). All analyses were performed using SPSS version 20 (IBM Corp., Armonk, NY, USA).

## References

[1] Bray F, Ferlay J, Soerjomataram I, Siegel RL, Torre LA, Jemal A (2018). Global cancer statistics 2018: GLOBOCAN estimates of incidence and mortality worldwide for 36 cancers in 185 countries. CA Cancer J. Clin..

[2] Lindenblatt RCR, Martinez GL, Silva LE, Faria PS, Camisasca DR, Lourenço SQC (2012). Oral squamous cell carcinoma grading systems—Analysis of the best survival predictor. J. Oral Pathol. Med..

[3] Liu S-A, Wang C-C, Jiang R-S, Lee F-Y, Lin W-J, Lin J-C (2017). Pathological features and their prognostic impacts on oral cavity cancer patients among different subsites—A singe institute’s experience in Taiwan. Sci. Rep..

[4] Baxi SS, Pinheiro LC, Patil SM, Pfister DG, Oeffinger KC, Elkin EB (2014). Causes of death in long-term survivors of head and neck cancer. Cancer.

[5] Mendel JR, Baig SA, Hall MG, Jeong M, Byron MJ, Morgan JC (2018). Brand switching and toxic chemicals in cigarette smoke: A national study. PLoS ONE.

[6] Archanjo AB (2020). Elemental characterization of oral cavity squamous cell carcinoma and its relationship with smoking, prognosis and survival. Sci. Rep..

[7] Valko M, Rhodes CJ, Moncol J, Izakovic M, Mazur M (2006). Free radicals, metals and antioxidants in oxidative stress-induced cancer. Chem Biol Interact..

[8] Reuter S, Gupta SC, Chaturvedi MM, Aggarwal BB (2010). Oxidative stress, inflammation, and cancer: How are they linked?. Free Radic. Biol. Med..

[9] Curtis CD, Thorngren DL, Nardulli AM (2010). Immunohistochemical analysis of oxidative stress and DNA repair proteins in normal mammary and breast cancer tissues. BMC Cancer.

[10] David SS, O’Shea VL, Kundu S (2007). Base-excision repair of oxidative DNA damage. Nature.

[11] Shah F, Logsdon D, Messmann RA, Fehrenbacher JC, Fishel ML, Kelley MR (2017). Exploiting the Ref-1-APE1 node in cancer signaling and other diseases: from bench to clinic. NPJ Precis Oncol..

[12] Powis G, Kirkpatrick DL (2007). Thioredoxin signaling as a target for cancer therapy. Curr. Opin. Pharmacol..

[13] Yanamoto S, Yamada S, Takahashi H, Yoshitomi I, Kawasaki G, Ikeda H (2012). Clinicopathological risk factors for local recurrence in oral squamous cell carcinoma. Int. J. Oral. Maxillofac. Surg..

[14] Jardim JF, Francisco ALN, Gondak R, Damascena A, Kowalski LP (2015). Prognostic impact of perineural invasion and lymphovascular invasion in advanced stage oral squamous cell carcinoma. Int. J. Oral Maxillofac. Surg..

[15] Jones HB, Sykes A, Bayman N, Sloan P, Swindell R, Patel M (2009). The impact of lymphovascular invasion on survival in oral carcinoma. Oral Oncol..

[16] Kim JM, Kim TY, Kim WB, Gong G, Kim SC, Hong SJ (2006). Lymphovascular invasion is associated with lateral cervical lymph node metastasis in papillary thyroid carcinoma. Laryngoscope..

[17] Close LG, Burns DK, Reisch J, Schaefer SD (1987). Microvascular invasion in cancer of the oral cavity and oropharynx. Arch. Otolaryngol. Neck. Surg..

[18] Michikawa C, Uzawa N, Kayamori K, Sonoda I, Ohyama Y, Okada N (2012). Clinical significance of lymphatic and blood vessel invasion in oral tongue squamous cell carcinomas. Oral Oncol..

[19] Matsushita Y, Yanamoto S, Takahashi H, Yamada S, Naruse T, Sakamoto Y (2015). A clinicopathological study of perineural invasion and vascular invasion in oral tongue squamous cell carcinoma. Int. J. Oral Maxillofac. Surg..

[20] Garavello W, Spreafico R, Gaini RM (2007). Oral tongue cancer in young patients: A matched analysis. Oral Oncol..

[21] Bonifazi M (2011). Age–period–cohort analysis of oral cancer mortality in Europe: The end of an epidemic?. Oral Oncol..

[22] Li Y, Mao Y, Zhang Y, Cai S, Chen G, Ding Y (2014). Alcohol drinking and upper aerodigestive tract cancer mortality: A systematic review and meta-analysis. Oral Oncol..

[23] Allen, N. *et al*. Alcohol Consumption and Ethyl Carbamate (ed. IARC Working Group on the Evaluation of Carcinogenic Risks to Humans) 237–329. (IARC Press, 2010). PMC4781168 21735939

[24] Nelson DE, Jarman DW, Rehm J, Greenfield TK, Rey G, Kerr WC (2013). Alcohol-attributable cancer deaths and years of potential life lost in the United States. Am. J. Public Health..

[25] Praud D, Rota M, Rehm J, Shield K, Zatoński W, Hashibe M (2016). Cancer incidence and mortality attributable to alcohol consumption. Int. J. Cancer..

[26] South AP (2019). Mutation signature analysis identifies increased mutation caused by tobacco smoke associated DNA adducts in larynx squamous cell carcinoma compared with oral cavity and oropharynx. Sci. Rep..

[27] Boyle JO (2010). Effects of cigarette smoke on the human oral mucosal transcriptome. Cancer Prevent. Res..

[28] Lin W-J (2011). Smoking, alcohol, and betel quid and oral cancer: A prospective cohort study. J. Oncol..

[29] Chen F (2019). Serum copper and zinc levels and the risk of oral cancer: A new insight based on large-scale case–control study. Oral Dis..

[30] Sachdev PK (2018). Zinc, copper, and iron in oral submucous fibrosis: A meta-analysis. Int. J. Dentistry..

[31] Khanna S (2013). Trace elements (copper, zinc, selenium and molybdenum) as markers in oral sub mucous fibrosis and oral squamous cell carcinoma. J. Trace Elements Med. Biol..

[32] Shi X, Dalal NS (1992). The role of superoxide radical in chromium(VI)-generated hydroxyl radical: The Cr(VI) haber-weiss cycle. Arch. Biochem. Biophys..

[33] Shi X, Dalal NS (1994). Generation of hydroxyl radical by chromate in biologically relevant systems: Role of Cr(V) complexes versus tetraperoxochromate(V). Environ. Health Perspect..

[34] Feng J, Xu J, Xu Y, Xiong J, Xiao T, Jiang C (2019). CLIC1 promotes the progression of oral squamous cell carcinoma via integrins/ERK pathways. Am. J. Transl. Res..

[35] Peretti M, Angelini M, Savalli N, Florio T, Yuspa SH, Mazzanti M (2015). Chloride channels in cancer: Focus on chloride intracellular channel 1 and 4 (CLIC1 AND CLIC4) proteins in tumor development and as novel therapeutic targets. Biochim. Biophys. Acta Biomembr..

[36] Yuzefovych LV, Kahn AG, Schuler MA, Eide L, Arora R, Wilson GL (2016). Mitochondrial DNA repair through OGG1 activity attenuates breast cancer progression and metastasis. Cancer Res..

[37] Kakehashi A, Ishii N, Okuno T, Fujioka M, Gi M, Fukushima S (2017). Progression of hepatic adenoma to carcinoma in Ogg1 mutant mice induced by phenobarbital. Oxid. Med. Cell Longev..

[38] Tell G, Damante G, Caldwell D, Kelley MR (2005). The intracellular localization of APE1/Ref-1: More than a passive phenomenon?. Antioxid. Redox Signal..

[39] Rani V, Deep G, Singh RK, Palle K, Yadav UCS (2016). Oxidative stress and metabolic disorders: Pathogenesis and therapeutic strategies. Life Sci..

[40] Benhar M, Engelberg D, Levitzki A (2002). ROS, stress-activated kinases and stress signaling in cancer. EMBO Rep..

[41] Kelley MR, Logsdon D, Fishel ML (2014). Targeting DNA repair pathways for cancer treatment: What’s new?. Future Oncol..

[42] Hornsveld M, Dansen TB (2016). The hallmarks of cancer from a redox perspective. Antioxid. Redox Signal..

[43] Welsh SJ, Bellamy WT, Briehl MM, Powis G (2002). The redox protein thioredoxin-1 (Trx-1) increases hypoxia-inducible factor 1α protein expression. Cancer Res..

[44] Kim WJ, Cho H, Lee S-W, Kim Y-J, Kim K-W (2005). Antisense-thioredoxin inhibits angiogenesis via pVHL-mediated hypoxia-inducible factor-1α degradation. Int. J. Oncol..

[45] Saitoh M, Nishitoh H, Fujii M, Takeda K, Tobiume K, Sawada Y (1998). Mammalian thioredoxin is a direct inhibitor of apoptosis signal-regulating kinase (ASK) 1. EMBO J..

[46] Cajaiba MM, Neves JI, Casarotti FF, de Camargo B, ChapChap P, Sredni ST (2006). Hepatoblastomas and liver development: A study of cytokeratin immunoexpression in twenty-nine hepatoblastomas. Pediatr. Dev. Pathol..

[47] Solé VA, Papillon E, Cotte M, Walter P, Susini J (2007). A multiplatform code for the analysis of energy-dispersive X-ray fluorescence spectra. Spectrochim. Acta Part B Spectrosc..

[48] Pérez CA, Radtke M, Sánchez HJ, Tolentino H, Neuenshwander RT, Barg W (1999). Synchrotron radiation X-ray fluorescence at the LNLS: beamline instrumentation and experiments. X-ray Spectrom..

[49] Soini Y, Kahlos K, Puhakka A, Lakari E, Säily M, Pääkkö P (2000). Expression of inducible nitric oxide synthase in healthy pleura and in malignant mesothelioma. Br. J. Cancer..

[50] dos Santos M, Mercante AMC, Louro ID, Gonçalves AJ, de Carvalho MB, da Silva EHT (2012). HIF1-alpha expression predicts survival of patients with squamous cell carcinoma of the oral cavity. PLoS ONE.

